# Fu’s subcutaneous needling therapy for intervertebral disk displacement: a systematic review and meta-analysis

**DOI:** 10.3389/fmed.2026.1781799

**Published:** 2026-04-13

**Authors:** Wei Tian, Xuemei Zheng, Peijie Qin, Shiheng Wang

**Affiliations:** 1 School of Traditional Chinese Medicine, Binzhou Medical University, Yantai, China; 2 Hengshui People’s Hospital (Harley Hospital International Peace), Hengshui, China; 3 Institute of Medical History and Literature, China Academy of Chinese Medical Sciences, Beijing, China; 4 Yunnan Provincial Hospital of Traditional Chinese Medicine, Kunming, China

**Keywords:** clinical efficacy, Fu’s subcutaneous needling, grade, intervertebral disk displacement, meta-analysis

## Abstract

**Background:**

Intervertebral disk displacement (IDD) significantly impairs quality of life and overall health. The incidence of IDD has been increasing due to rising social pressures. Fu’s subcutaneous needling, an advanced acupuncture technique, demonstrates several advantages over traditional filiform needles. However, its clinical efficacy remains a subject of debate. This study employed a meta-analysis approach to comprehensively evaluate the therapeutic efficacy of Fu’s subcutaneous needling for IDD.

**Methods:**

A systematic search was conducted across multiple databases, such as PubMed, EMbase, Cochrane Library, Web of Science, CNKI database, Wanfang database, VIP database, and Chinese Biomedical Literature Service System (CBM), for randomized controlled trials (RCTs) on Fu’s subcutaneous needling therapy for IDD published up to 10 March 2025. Meta-analysis was performed using RevMan 5.4 software.

**Results:**

A total of 32 RCTs meeting the criteria were finally included, involving 2,778 subjects. The meta-analysis evidence indicated that the clinical total effective rate of Fu’s subcutaneous needling group was significantly higher than that of the control group (RR = 1.16, 95% CI [1.11, 1.20], *p* < 0.05, *I*^2^ = 50%). In terms of pain relief: (VAS score: MD = −1.69, 95% CI [−2.59, −0.78], *p* < 0.05, I2 = 99%), improvement in lumbar function (JOA score: MD = 3.97, 95% CI [2.61, 5.34], *p* < 0.05, I2 = 92%), and (ODI index: MD = −7.50, 95% CI [−8.79, −6.21], *p* < 0.05, I2 = 92%), the Fu’s subcutaneous needling group demonstrated superior intervention effects in all aspects. Subgroup analysis showed that different control measures (such as filiform needles and electroacupuncture) and treatment courses might be sources of heterogeneity. The GRADE evaluation indicated that, due to high heterogeneity and sample size limitations, the quality of evidence for the main outcome was moderate to very low. Sensitivity analysis suggested that the combined effect value was stable, and the publication bias assessment indicated that the possibility of significant bias was low.

**Conclusion:**

Fu’s subcutaneous needling therapy demonstrates good efficacy and safety for IDD. Although this study supports its clinical application, the quality of the included studies is relatively low. Therefore, more high-quality, multicenter RCTs are warranted in the future to further validate its therapeutic efficacy.

**Systematic review registration:**

https://www.crd.york.ac.uk/PROSPERO/view/CRD420251001904.

## 1 Introduction

Intervertebral disk displacement (IDD) is a common degenerative spinal condition and a frequent cause of low back and leg pain (1). Symptoms include lower back pain, radiating leg pain, leg numbness, muscle weakness, and even bowel and bladder dysfunction (2). These symptoms significantly reduce patients’ quality of life, impairing their ability to work, study, and perform daily activities, thereby affecting family happiness and increasing societal burden. With societal advancements and changes in work habits, the incidence of IDD is increasingly affecting younger populations, making timely and effective prevention and treatment crucial (3).

Traditional treatments for IDD include surgical and conservative approaches. Some patients experience significant relief with conservative treatments such as physical therapy or medication. However, there is a lack of specific drugs for IDD, and current medications primarily focus on anti-inflammatory, blood-activating, and nerve-nourishing effects, offering limited therapeutic benefits. Many patients do not improve with conservative treatments and eventually require surgery (4). Traditional surgical methods are invasive, carry high risks of postoperative infection and significant blood loss, and can lead to complications such as scarring, adhesions, and spinal instability (5). In recent years, minimally invasive surgical techniques have gradually replaced traditional surgical methods for IDD.

Fu’s subcutaneous needling (FSN), developed by Professor Fu Zhonghua in 1996, is a novel external therapy involving subcutaneous stimulation to treat musculoskeletal conditions (6). The mechanism of action involves the repeated pulling of subcutaneous connective tissue, which rapidly relieves muscle spasms, improves blood supply and nutrition to the affected area, and reduces local inflammation (7). Although its exact mechanism remains unclear, studies have shown its effectiveness in treating IDD. This study aims to evaluate the efficacy of FSN for IDD through meta-analysis, providing evidence-based medical support (the introduction of FSN is in the Supplementary materials).

## 2 Methods

### 2.1 Registration

This research has been registered in https://www.crd.york.ac.uk/PROSPERO/myprospero: CRD420251001904.

### 2.2 Inclusion criteria

#### 2.2.1 Study design

A randomized controlled trial.

#### 2.2.2 Participants

Patients clearly diagnosed with intervertebral disk displacement, as the diagnostic criteria used for inclusion studies were not uniform, could be included as long as it was clearly stated that they met the diagnostic criteria. There were no restrictions on the nationality, age, gender, or race of the patients.

#### 2.2.3 Intervention

Fu’s subcutaneous needling: there is no limit to the course of treatment. Comparison: conventional treatment.

#### 2.2.4 Outcome

Primary outcome measure: (1) Efficacy (8). The main reference for inclusion in the study was the “Standards for Diagnosis and Therapeutic Effect of Traditional Chinese Medicine Syndromes and Diseases.” According to the statistical method formula of nimodipine, the improvement index = [((JOA score after treatment - JOA score before treatment) ÷ JOA score before treatment) × 100%]. Cure: pain and other symptoms basically disappear, and lumbar movement is normal, with an improvement index of ≥ 90.00%; marked effect: pain and other symptoms have significant improvement, with mild restriction in lumbar movement, with an improvement index of 66.67% to < 90.00%; improvement: pain and other symptoms have slightly improved, but lumbar movement is still restricted, with an improvement index of 33.33% to < 66.67%; ineffective: there is no significant improvement in symptoms, with an improvement index < 33.33%. Efficacy = (Number of cured cases + Number of effective cases + Number of improved cases)/total number of cases × 100%; (2) visual analog scales (VAS) score (9): this is used to assess the intensity of pain. The score ranges from 0 to 10; the higher the score, the greater the degree of pain. (3) Adverse reaction. Secondary outcome measures: (1) Japanese Orthopaedic Association (JOA) (10): A scoring system used to evaluate human functional disorders, primarily used to assess the efficacy of treatment for low back pain disorders ‌. The total score was up to 29 and down to 0. The lower the score, the more pronounced the dysfunction. (2) Oswestry Disability Index (ODI) (11): A scale used to assess low back pain dysfunction. The total score ranges from 0 to 50, with higher scores indicating more severe dysfunction.

### 2.3 Exclusion criteria

Republished studies; non-randomized controlled trials (conference abstracts, reviews, case reports, etc.); interventions do not fit; studies with no access to the full text; and incomplete data.

### 2.4 Data sources and search strategy

Search for randomized controlled trials on Fu’s subcutaneous needling therapy for intervertebral disk displacement is published in PubMed, EMbase, Cochrane Library, Web of Science, CNKI database, Wanfang database, VIP database, and Chinese Biomedical Literature Service System (CBM) up to 10 March 2025 (Table 1; the search strategies for the remaining databases are provided in the Supplementary materials).

**Table 1:** PubMed search strategy.

**Search number**	**Query**
#1	Fu’s subcutaneous needling OR Fu’s acupuncture therapy OR float needle[Title/Abstract]
#2	Intervertebral Disc Displacement[MeSH Terms]
#3	Intervertebral Disc Displacement* OR Protruded Disc* OR Protruded Disk* OR Intervertebral Disk Displacement* OR Herniated Disk* OR Slipped Disk* OR Disk Prolapse* OR Prolapsed Disk* OR Herniated Disc* OR Slipped Disc* OR Prolapsed Disc* OR Disc Herniation* OR Disk Herniation* OR Disc Protrusion* OR Disk Protrusion*[Title/Abstract]
#4	#2 OR #3
#5	#1 AND #4

### 2.5 Study selection

Two researchers independently conducted the literature screening process in accordance with the predefined search strategy. The retrieved articles were then imported into EndNote X9 software for further management and screening. Initially, the titles and abstracts of all articles were independently reviewed to exclude studies that clearly failed to meet the inclusion criteria. Subsequently, for potentially eligible studies, the full texts were examined to confirm their compliance with the inclusion criteria. In cases of disagreement, consensus was achieved through discussion or, if necessary, arbitration by a third party.

### 2.6 Data extraction

Two researchers independently conducted the literature screening process in accordance with the predefined search strategy and systematically documented the relevant information using customized data extraction tables. The following data categories were extracted: (1) general information: author, publication year, sample size, intervention details, control group measures, follow-up duration, etc.; (2) efficacy-related data: values of primary and secondary outcome measures; (3) quality assessment information: methodological characteristics of the study and risk of bias.

### 2.7 Risk of bias in studies

The included literature was evaluated for quality and risk of bias using the risk of Bias assessment tool (12) recommended in the Cochrane Handbook 5.3.0. The assessment criteria included whether random methods were used for allocation, allocation concealment, practitioner, subject, outcome evaluation blindness, completeness of outcome data, selective results, and other sources of bias. Each item was classified as “low risk,” “high risk,” and “unclear.”

### 2.8 Statistical analysis

Statistical analysis was performed using Revman5.4. First, the heterogeneity test was conducted: the *Q*-value statistic test was used for the test, the fixed effects model was used when *p* ≥ 0.1 or I2 ≤ 50%, and the random effects model was used otherwise. If heterogeneity is high, subgroup analysis was used, etc., to find the source of heterogeneity. Count data were estimated using relative risk (RR) and its 95% confidence interval, while measure data were weighted and combined using mean difference (MD) and its 95% confidence interval. The test level *α* was defined as 0.05, and *p* < 0.05 was considered statistically significant. According to the Cochrane manual, publication bias was judged and analyzed using funnel plots and Egger’s regression plots if the number of studies included in a single outcome measure was ≥10. Sensitivity analyses were performed to examine the stability of the results. If heterogeneity is significant (*I*^2^ > 50%), subgroup analysis will be conducted to identify the sources. Based on the characteristics of clinical heterogeneity, the preset subgroup analyses include: types of control measures, treatment duration, etc.

### 2.9 GRADE evidence quality assessment

The GRADE System (13) was used for evidence quality assessment. The GRADE system includes five categories: limitations, inconsistencies, indirectness, imprecision, and publication bias, and grades the evidence quality of the study results into four levels: high, medium, low, and very low. Two researchers completed the assessment separately, and a third party would decide in case of dispute. Use GRADEpro to create quality maps of evidence.

## 3 Results

### 3.1 Study selection

**Figure 1:**
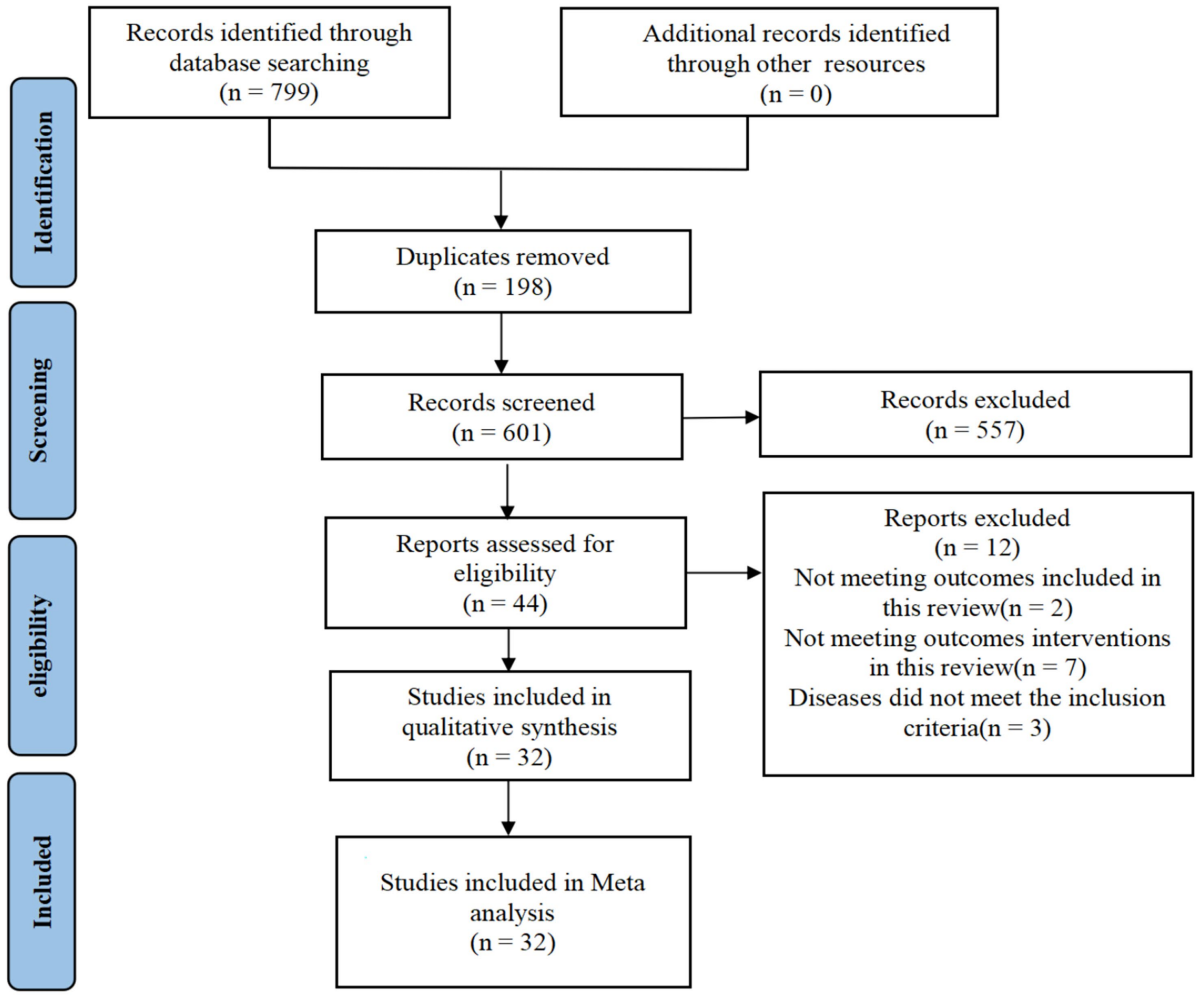
Flowchart of literature screening (PRISMA).

A total of 799 articles were retrieved, 198 duplicate articles were excluded, 557 bibliographies and abstracts were excluded, 12 full texts were excluded from reading and downloading, and, finally, 32 randomized controlled trials were included (Figure 1).

### 3.2 Study characteristics

A total of 32 studies were included (14–45), with a total sample size of 2,778 cases (Fu’s subcutaneous needling group: 1,390 cases, control group: 1,388 cases). The sample size range of the treatment group was 30–90 cases, and that of the control group was also 30–90 cases. The treatment course range was 7–60 days. Table 2 is the basic information table of the included literature.

**Table 2:** Basic information table of included literature.

**Author**	**Publication years**	**Intervention methods**	**Number of cases**	**Age**	**Course of treatment**	**Outcome indicators**
Xu Huaming (14)	2006	AvsB	51 ploidy	45	21d	Efficacy
Qin He (15)	2016	AvsC	40,40	60.35 ± 8.82, 57.80 ± 7.14	10d	Efficacy
Wang Yingjie (16)	2016	AvsB	50, 50	40.0 ± 4.1, 39.0 ± 5.2	14d	Efficacy, AE
Peng Jiping (17)	2019	AvsB	60, 60	49.65 ± 3.52, 47.16 ± 5.56	7d	Efficacy
Yang Yongguang (18)	2015	AvsB	90, living	25–70	10d	Efficacy
Yuan Zhiqi (19)	2018	AvsB	47 and	38.64 ± 5.31, 38.55 ± 5.43	14d	Efficacy
Yuan Xingxing (20)	2017	AvsC	80,80	43.60 ± 6.24, 40.78 ± 6.67	10d	Efficacy
Li Wanying (21)	2020	AvsC	32, 32	32.78 ± 7.67, 38 ± −6.68	14d	Efficacy, VAS
Li Jianhua (22)	2020	AvsB	42 and	36.14 ± 5.37, 36.22 ± 6.01	21d	Efficacy
Yang Jiangxia (23)	2020	AvsB	40,40	49.00 ± 1.96, 49.00 ± 1.96	21d	Efficacy, VAS, ODI, JOA
Huang Saizhi (24)	2015	AvsC	51, 50	40.1 ± 6.7, 40.5 ± 5.4	10d	Efficacy
Sun Chuang (25)	2019	AvsB	38, 38	41.4 ± −6.2, 41.5 ± 6.5	21d	Efficacy
Li Changsheng (26)	2011	AvsB	46, 50	45	30d	Efficacy
Xiang Changyun (27)	2015	AvsB	40,40	32 ± 3.4	10d	Efficacy
Zhang Jichen (28)	2011	AvsB	40,40	41 ± 8, 38 ± 6	10d	Efficacy
Huang Yucong (29)	2019	AvsB	40,40	52.31 ± 10.24, 49.35 ± 10.25	10d	Efficacy
Zeng Fangang (30)	2024	AvsE	30, 30	32.91 ± −4.37, 33.08 ± 4.45	60d	Efficacy, VAS, JOA
Chen Chunli (31)	2022	AvsB	30, 30	40.2 ± 8.3, 40.8 ± 7.8	14d	Efficacy, VAS,
Chen Wenjing (32)	2023	AvsB	41 9	41.85 ± 5.76, 41.37 ± 5.83	21d	Efficacy, ODI, AE
Chen Yunyao (33)	2024	AvsD	40,40	35.20 ± 8.04, 35.85 ± 8.16	14d	Efficacy, VAS, ODI
Cheng Hui (34)	2024	AvsD	30, 30	43.30 ± 13.77, 43.1313.20	21d	VAS, ODI
Fan Shaoting (35)	2024	AvsB	32, 32	49.78 ± 8.25, 50.28 ± 9.03		Efficacy, VAS, JOA, AE
Hu Chaying (36)	2025	AvsF	35, 35	37.49 ± 5.21, 36.95 ± 5.35	21d	Efficacy, VAS, JOA
Jia Ming (37)	2023	AvsC	50, 50	55.8 ± 4.2, 56.6 ± 3.8	10d	Efficacy, VAS, JOA
Li Bowen (38)	2023	AvsB	38, 38	39.70 ± 3.13, 40.164.09	14d	Efficacy, VAS, ODI
Li Hongpeng (39)	2024	AvsB	50, 50	53.42 ± 9.15, 52.96 ± 9.18		VAS
Meng Xianyu (40)	2023	AvsB	30, 30	54.70 ± 17.29, 52.10 ± 13.12	21d	Efficacy, ODI, JOA
Wang Ling (41)	2023	AvsB	39 July	40.61 ± 3.65, 40.78 ± 2.45	14d	Efficacy, ODI
Xiao Ju (42)	2024	AvsB	30, 30	53.98 ± 10.11, 53.78 ± 10.78	14d	Efficacy, ODI
Yuan Baohua (43)	2024	AvsB	35, 35	23.63 ± 5.12, 24.57 ± 5.52	14d	VAS, ODI
Zhao Qi (44)	2024	AvsB	60, 60	46.89 ± 5.87, 47.09 ± 5.96	28d	Efficacy, ODI, VAS
Zheng Liming (45)	2023	AvsF	33,33	38.8 ± 7.6, 38.1 ± 6.2	28d	JOA, ODI, VAS

A: Fu’s subcutaneous needling; B:filiform needle; C: electroacupuncture; D: medicine; E: shock wave; F: recovery.

### 3.3 Risk of bias instudies

**Figure 2:**
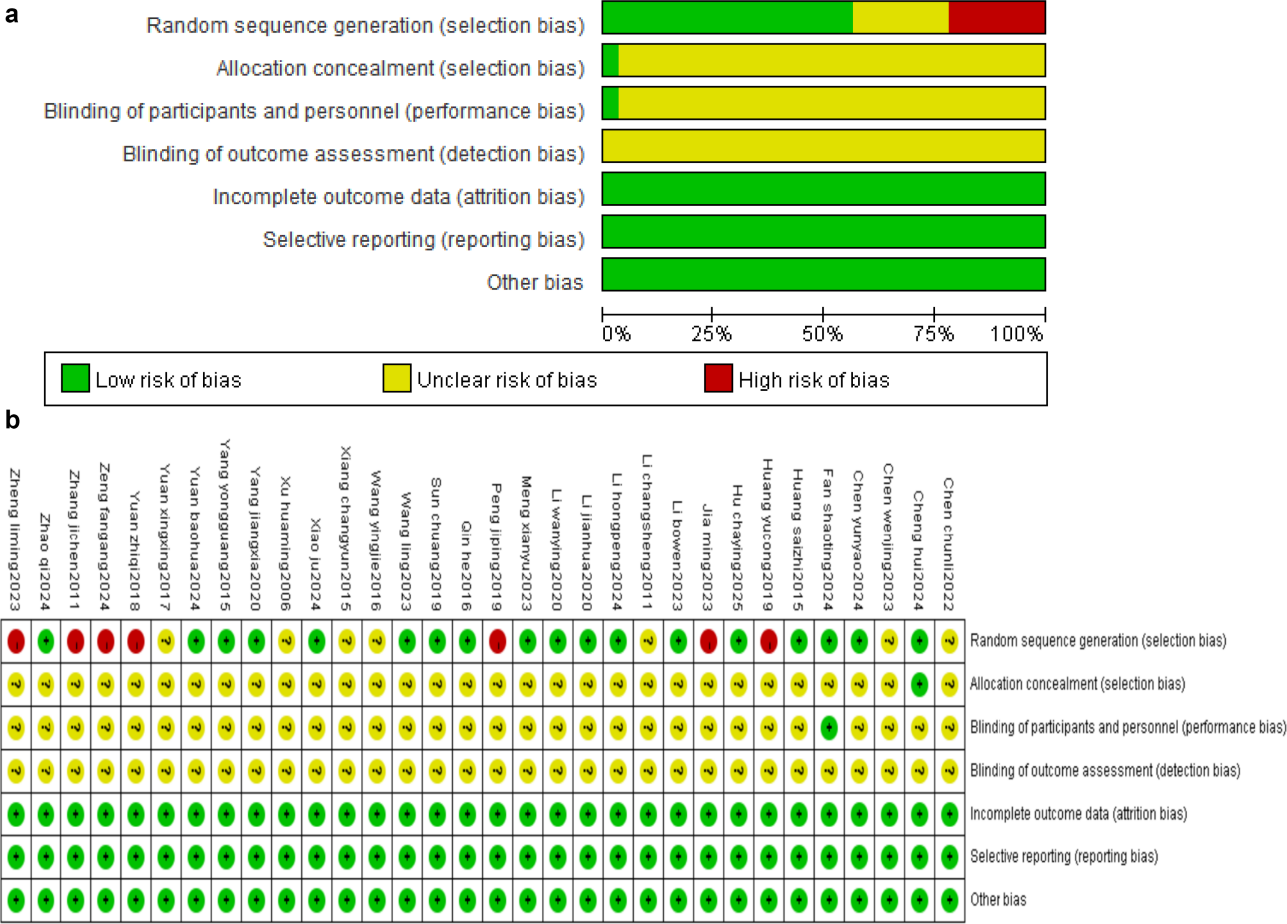
**(a)** Published bias assessment results. **(b)** Publication of bias evaluation results.

In terms of randomization methods, 18 studies used the low-risk randomization method, and 7 studies used the high-risk randomization method. In terms of assignment concealment, one study used envelopes. In terms of blinding, one study (40) used a blind method. Blinding was difficult due to the nature of Fu’s subcutaneous needling, though it had little effect on the results. Data from all studies were complete, and no sources of selective reporting and other publication biases were found. Risk of bias results indicated an average overall quality of the studies. Eighteen studies were “low risk,” 56%; 7 studies were “high risk,” 22%; and 7 studies were “unclear risk,” 22% (Figures 2a,b).

### 3.4 Meta analysis

#### 3.4.1 Efficacy

**Figure 3:**
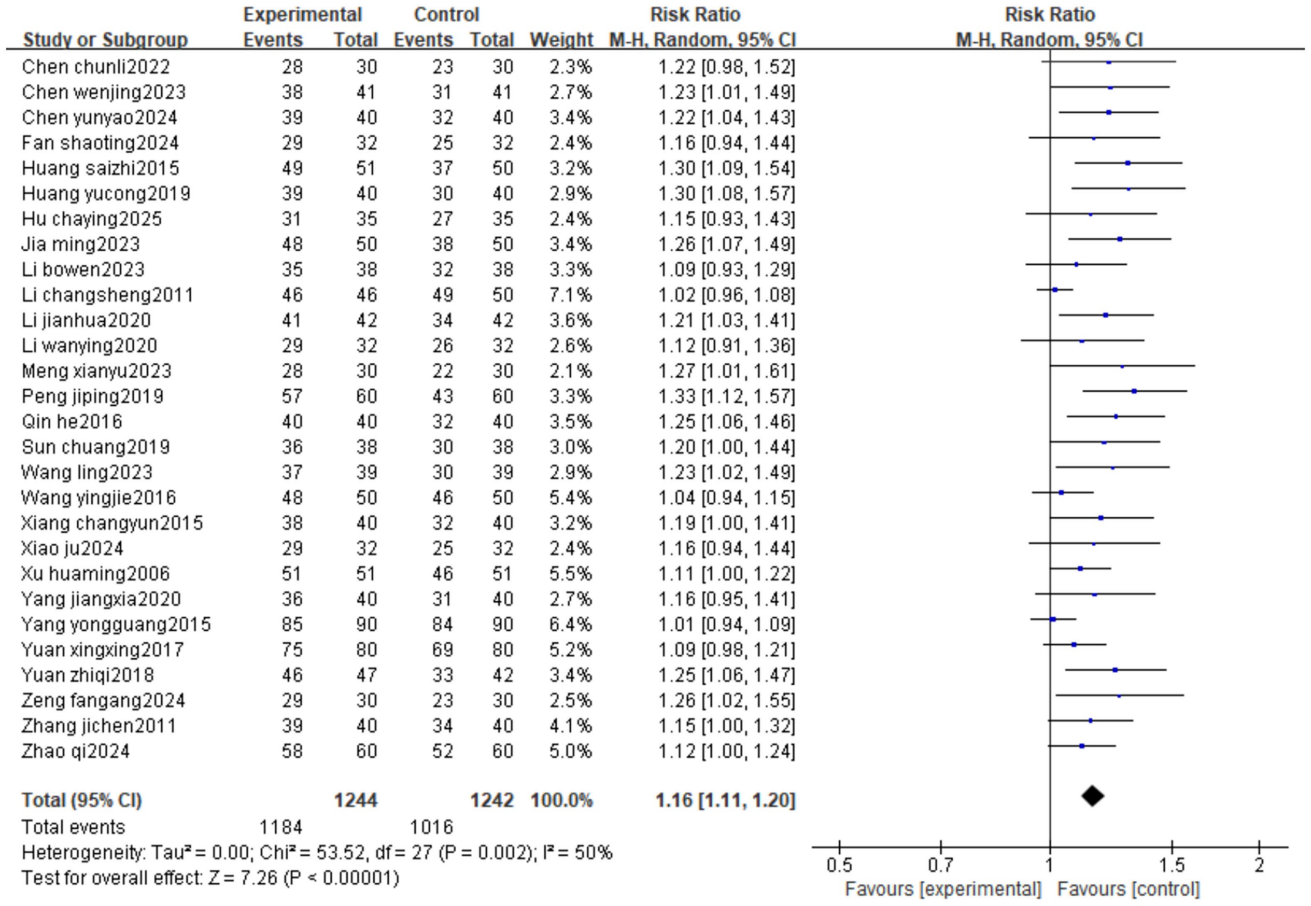
An efficacy forest image of Fu’s subcutaneous needling therapy for IDD.

Twenty-eight studies reported efficacy. The heterogeneity test result was *I*^2^ = 52%, suggesting moderate heterogeneity, using a random-effects model. The results of the meta-analysis showed that the intervention group had a higher efficacy than the control group (RR = 1.16, 95%CI[1.11, 1.20]) (Figure 3).

#### 3.4.2 Visual analog scales

**Figure 4:**
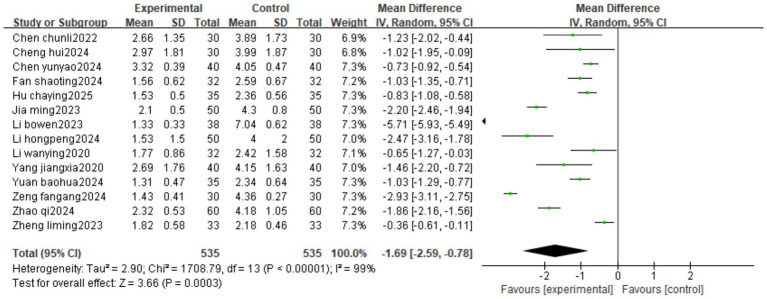
Forest image of VAS for Fu’s subcutaneous needling treatment.

Fourteen studies reported VAS. The heterogeneity test result was I^2^ = 99%, suggesting high heterogeneity, using a random-effects model. The results of the meta-analysis showed that the intervention group had a better effect in reducing VAS than the control group (MD = –1.69, 95%CI[−2.59, −0.78]) (Figure 4).

#### 3.4.3 Japanese orthopaedic association

**Figure 5:**
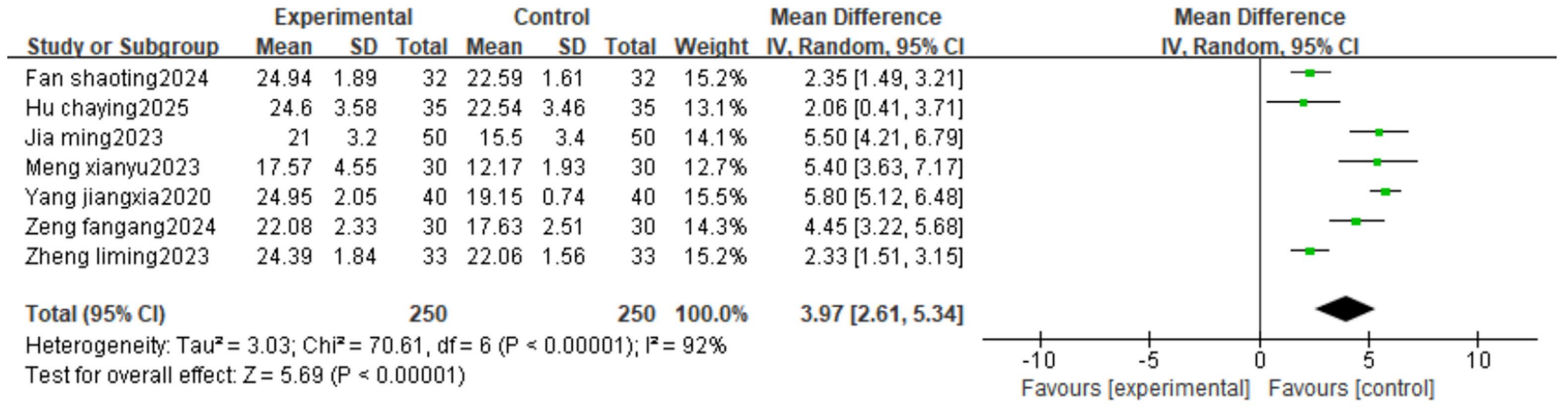
Forest image of JOA treated with Fu’s subcutaneous needling for IDD.

Fourteen studies reported JOA. The heterogeneity test result was *I*^2^ = 92%, suggesting high heterogeneity, using a random-effects model. The results of the meta-analysis showed that the intervention group had a better effect in reducing JOA than the control group (MD = 3.97, 95%CI[2.61, 5.34]) (Figure 5).

#### 3.4.4 Oswestry disability index

**Figure 6:**
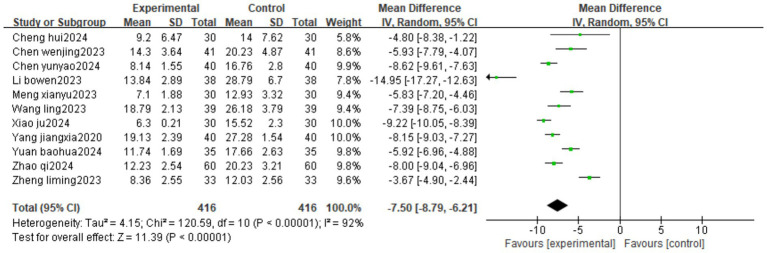
Forest plot of ODI treated with Fu’s subcutaneous needling for IDD.

Eleven studies reported ODI. The heterogeneity test result was *I*^2^ = 92%, suggesting high heterogeneity, using a random-effects model. The results of the meta-analysis showed that the intervention group was more effective in reducing ODI than the control group (MD = −7.50, 95%CI[−8.79, −6.21]) (Figure 6).

#### 3.4.5 Subgroup analysis

The subgroup analysis results are shown in Table 3. After stratification by the control measures, the heterogeneity of the effective rate in Fu’s subcutaneous needling vs. electro-acupuncture (AvsC) subgroup was significantly lower compared to the overall results (*I*^2^ = 41%), suggesting that the type of control measures is an important factor influencing the heterogeneity of the effective rate. However, for the VAS and ODI indicators, even when stratified by control measures or treatment courses, there was still a high degree of heterogeneity within each subgroup (*I*^2^ was generally > 85%). This indicates that in addition to the control measures and treatment courses, the baseline pain level of patients, the duration of the disease, the details of the Fu’s subcutaneous needling operation (such as whether reperfusion activities are uniformly carried out), and the differences in the assessment time points of the scales may be the more significant sources causing the high heterogeneity of continuous indicators. Due to the limitations of the original research data, this study was unable to conduct a more in-depth quantitative meta-analysis of these factors (Table 3).

**Table 3:** Results of the subgroup analysis of Fu’s subcutaneous needling treatment.

**Outcomes**	**Subgroup**	**Efficacy**			**VAS**			**ODI**		
		**No**	**RR (95%CI)**	***I*** ^**2**^	**No**	**MD (95%CI)**	***I*** ^**2**^	**No**	**MD (95%CI)**	***I*** ^**2**^
Intervention	AVSB	20	1.15 (1.10, 1.21)	55%	7	2.12 (3.87, 0.37)	99%	8	8.03 (9.39, 6.67)	90%
	AVSC	5	1.18 (1.09, 1.28)	41%						
Time	10d	8	1.17 (1.08, 1.27)	63%						
	14d	8	1.16 (1.08, 1.24)	0%				5	9.01 (10.99, 7.03)	89%
	21d	7	1.16 (1.09, 1.24)	0%				4	6.49 (8.10, 4.88)	75%

#### 3.4.6 Sensitivity analysis

Sensitivity analyses were performed for each outcome measure. The results of the sensitivity analysis showed that the results of the meta-analysis were stable (Table 4).

**Table 4:** Results of the sensitivity analysis of Fu’s subcutaneous needling therapy for IDD.

**Outcomes**	**Meta analysis**	**Sensitivity analysis**
Efficacy	1.15 (1.12, 1.21)	1.15 (1.11, 1.21)
VAS	1.69 (2.59, 0.78)	1.68 (2.58, 0.78)
JOA	3.97 (2.61, 5.34)	3.97 (2.60, 5.34)
ODI	7.50 (8.79, 6.21)	7.50 (8.79, 6.20)

#### 3.4.7 Publication bias

**Figure 7:**
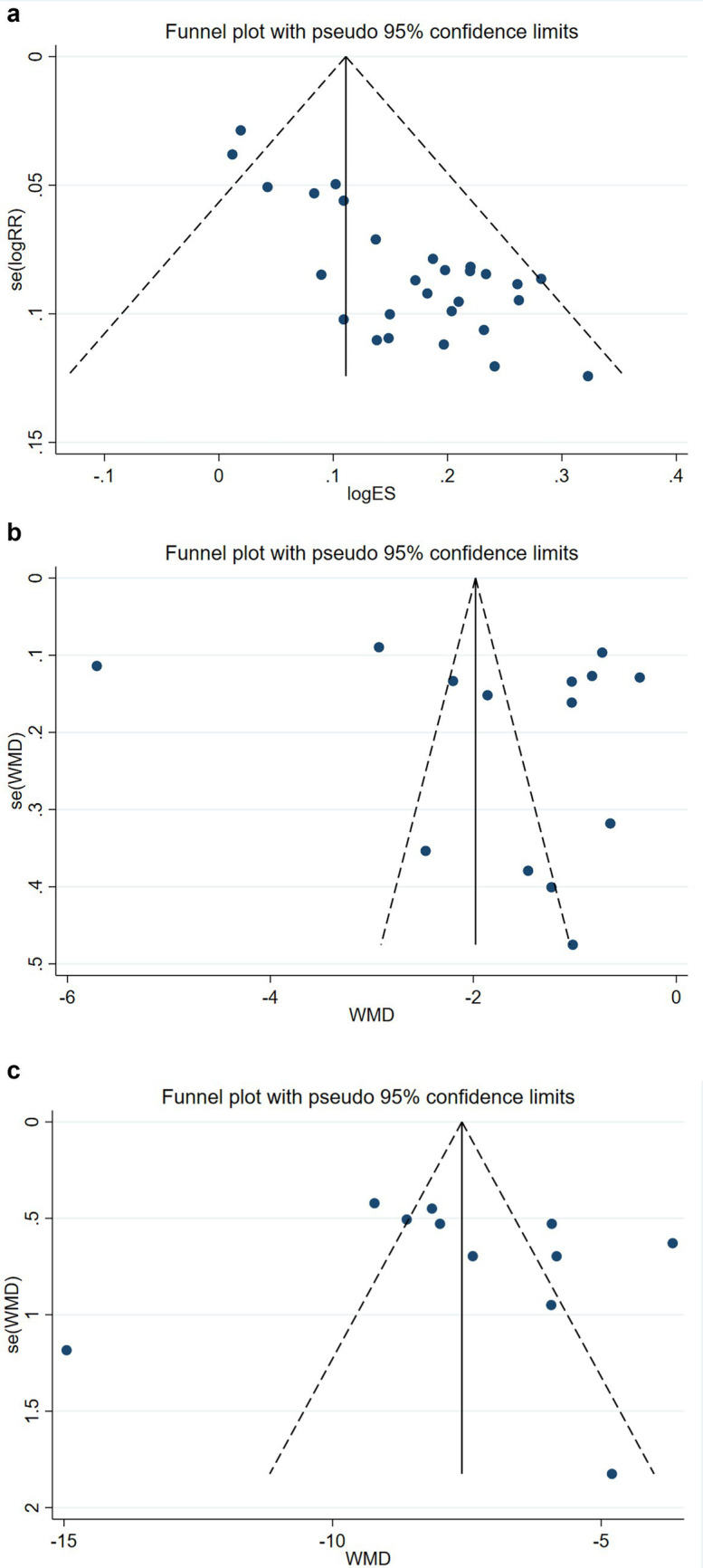
**(a)** An efficacy publication bias funnel plot of Fu’s subcutaneous needling therapy for IDD. **(b)** Publication bias funnel plot of VAS for Fu’s subcutaneous needling treatment of IDD. **(c)** Publication bias funnel plot of ODI treated with Fu’s subcutaneous needling for IDD.

Publication bias analyses were conducted for efficacy, VAS, and ODI. Egger’s test results showed *p* > 0.05, and the funnel plot indicated a low likelihood of publication bias (Figure 7).

#### 3.4.8 GRADE evidence quality assessment

GRADE evidence quality assessment was conducted on outcome measures, and the results showed that the main factor for downgrading was high heterogeneity. There were three high-quality pieces of evidence, accounting for 23%. There were three moderate-quality pieces of evidence, accounting for 23%. There were six pieces of low-quality evidence, accounting for 46%. There was one piece of evidence of very low quality, accounting for 8%. Overall quality is average (Table 5).

**Table 5:** Quality of GRADE evidence for Fu’s subcutaneous needling therapy in IDD.

**Outcome**	**Subgroup**	**No of studies**	**Risk of bias**	**Inconsistency**	**Indirectness**	**Imprecision**	**Publication bias**	**Sample size**		**Effect**	**Quality**
								**Intervention**	**Control**		
Efficacy		27	–	1 (1)	–	–	–	2,155	2,147	1.15 (1.12, 1.19)	Moderate
	10d	8	–	1 (1)	–	–	–	431	430	1.17 (1.08, 1.27)	Moderate
	14d	8	–	–	–	–	–	308	308	1.14 (1.07, 1.20)	High
	21d	7	–	–	–	–	–	277	277	1.16 (1.09, 1.24)	High
	AvsB	20	–	1 (1)	–	–	–	886	885	1.15 (1.09, 1.20)	Moderate
	AvsC	5	–	–	–	–	–	253	252	1.18 (1.09, 1.28)	High
VAS		11	–	–2 (1)	–	–	–	603	603	2.08 (0.91, 3.26)	Low
	AvsB	7	–	–2 (1)	–	–	–	285	285	2.12 (0.37, 3.87)	Low
ODI		10	–	–2 (1)	–	–	–	636	636	7.95 (7.05, 8.86)	Low
	AvsB	8	–	–2 (1)	–	–	–	313	313	8.03 (6.67, 9.39)	Low
	14d	5	–	–2 (1)	–	–	–	182	182	9.01 (7.03, 10.99)	Low
	21d	4	–	–2 (1)	–	1 (2)	–	141	141	6.49 (4.88, 8.10)	Very low
JOA		7	–	–2 (1)	–	Low	–	250	250	3.97 (2.61, 5.34)	Low

Downgrading factors: (1) 50% < *I*^2^ < 75% downgrade by 1 level; *I*^2^ ≥ 75% downgrading by 2 levels; (2) Downgrade 1 if the total sample size is less than 300 cases.

#### 3.4.9 Adverse reaction

Three studies reported adverse events. In the FSN group, the most frequent event was subcutaneous bleeding (five cases), followed by tachycardia (one case), skin itching (one case), and needling sticking (one case). In the control group, events included pain (three cases), bleeding (four cases), tachycardia (one case), and gastrointestinal reaction (one case). All events were mild and self-limiting, with no severe adverse events reported. The comparable safety profile between groups further supports the clinical applicability of FSN. However, due to inconsistent reporting across studies, the safety evidence should be interpreted with caution, and future trials should adopt standardized adverse event reporting (Table 6).

**Table 6:** Adverse reaction.

**Study**	**Intervention group**	**Control group**
Wang Yingjie (14)	Bleeding (3 cases)	Pain (3 cases)
Chen Wenjing (32)	Bleeding (1 case), sticking of needle (1case), infection at the acupuncture site (1 case)	Bleeding (2 cases), sticking of needle (1case), infection at the acupuncture site (2 cases)
Fan Shaoting (35)	skin itch (1 case), tachycardia (1 case), bleeding (1 case),	tachycardia (1 case), gastrointestinal reaction (1 case), bleeding (2 cases)

## 4 Discussion

The purpose of this study was to explore the evidence-based evidence for the clinical efficacy of Fu’s subcutaneous needling therapy for IDD. A total of 32 studies, involving 2,778 patients, were included. Efficacy, VAS, JOA, and NDI were studied. The results showed significant advantages of Fu’s subcutaneous needling therapy for IDD. However, there was greater heterogeneity in certain outcome measures. We conducted subgroup analyses and found that Fu’s subcutaneous needling had an advantage over other treatments. However, due to the small number of studies included in the subgroup analysis, the results need to be further confirmed. The results of the sensitivity analysis showed that the results of the meta-analysis were stable. Publication bias results showed a low likelihood of publication bias. However, due to the small number of studies included, the results need to be further verified. The GRADE evidence assessment results showed that the quality of the evidence was moderate.

The Fu’s subcutaneous needling technique was invented in 1996 by Professor Fu Zhonghua from Guangzhou University of Chinese Medicine as an improved filiform needle method based on traditional acupuncture. In this study, the effect of the Fu’s subcutaneous needling group was significantly better than that of the fine needle group, which was reflected in the immediate effect of the Fu’s subcutaneous needling. In terms of immediate pain relief (VAS), Fu’s subcutaneous needling group was more effective than the filiform needle group. In terms of long-term efficacy, Fu’s subcutaneous needling group was slightly better than the filiform needle group (as shown in efficacy, VAS, and ODI scores). The reason for this difference is to first consider the possible mechanisms of action of both sides. In traditional needling, acupoints are selected based on the theory of meridians in traditional Chinese medicine. By stimulating the acupoints, the needling can increase the release of opioid peptides and serotonin in the neurotransmitters in the brain, activate the norepinephrine ascending projection system in the brain, activate the norepinephrine descending projection system from the lower brainstem, and the central acetylcholinergic system can impede the conduction of pain (46, 47). Accompanying the study of the neurochemical mechanism of filiform needle analgesia is the induction period of filiform needle analgesia (the analgesic effect requires at least 20–40 min of induction time) (48). Skin tissue plays a crucial role in the peripheral modulation of pain perception. Nerves in the peripheral mechanisms of pain modulation. Within the skin tissue, a new type of endogenous neurotransmitter called endocannabinoid can be synthesized. This is very similar to the benign and bidirectional adjustment function that the body has for its own functional activities. A new type of neurotransmitter, endocannabinoids, can be synthesized within the skin tissue, which is very similar to the benign bidirectional regulation of the body’s functional activities by the needle (49).

**Figure 8:**
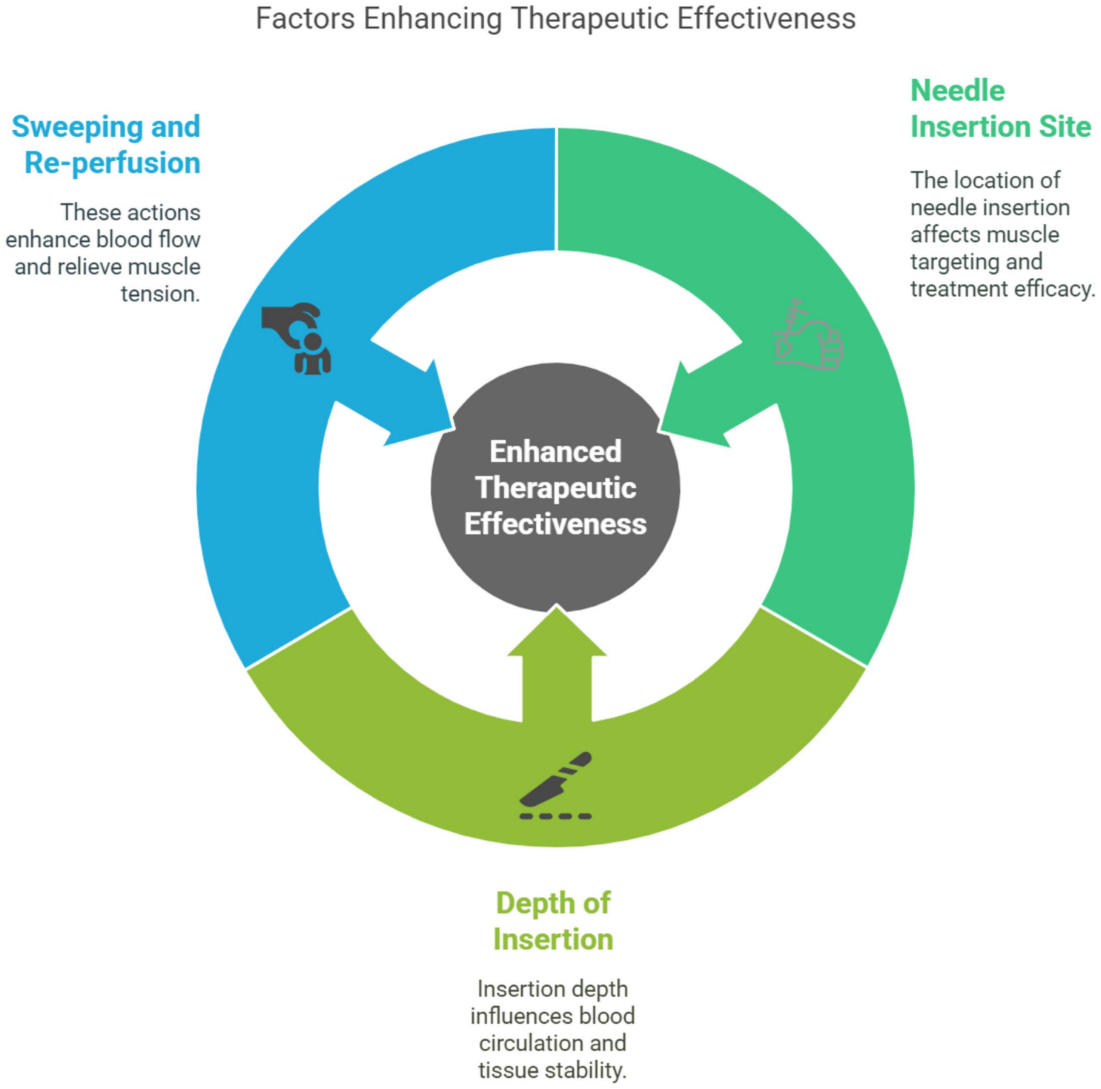
Advantages of Fu’s subcutaneous needling over filiform needle.

The guiding theory of the Fu’s subcutaneous needling, which inherits the theory of traditional Chinese medicine and integrates the theory of modern medicine, is more advanced in concept. The advantages of Fu’s subcutaneous needling are mainly reflected in several aspects. First, the place where the needle is inserted. The site of the needle insertion has a significant impact on (myofascial trigger points, MTrP). The Fu’s subcutaneous needling is chosen with the tip directly facing the affected muscle. When the number of affected muscles is small and concentrated, near-needle insertion is used; when the number of affected muscles is large, long-range bombing is generally adopted. When choosing the affected muscle, it is also important to note that the erector spinae should not only be treated on the affected side but also on the healthy side. If the affected muscle appears, the muscle strength will be unbalanced, and only treating the affected side will have limited efficacy; the iliopsoas and oblique abdominal muscles should not be ignored. Some patients experience increased pain when sneezing, considering that sneezing pulls the related muscles and causes increased pain. When patients experience pain on the anterior or lateral side of the lower leg, the tibialis anterior muscle or the peroneus longus muscle can be treated with a catheter, which is highly effective (50). Second, the depth of the needle insertion. The needle is inserted into the shallow fascia layer. This layer of tissue channels has the functions of transmitting information, matter, energy, cleaning the channels, and maintaining the stability of the microenvironment. Unblocking the tissue channels creates the necessary conditions for maintaining the stability of the microenvironment (51). Compared with the needle, Fu’s subcutaneous needling improves local blood circulation more directly. Third, the biggest difference between Fu’s subcutaneous needling and traditional acupuncture is the sweeping and reperfusion activity of Fu’s subcutaneous needling. Sweeping is an action carried out from the moment the needle is inserted until it is removed, and it can be divided into flat sweeping and rotary sweeping. After the needle is inserted, the body of the needle is pressed down, the tip of the needle points to the affected area, and the handle of the needle is slowly swung left and right to gradually spread the sensation of the needle. Therefore, sweeping also has the effect of warming and promoting the flow of qi and blood. From the perspective of Western medicine anatomy, Fu’s subcutaneous needling enters the superficial fascia layer. When it is swept, the local tissue channels are relatively unobstructed. Due to the effects of bioelectricity, chemical substances, etc., muscle spasms are relieved, blood vessels are relatively dilated, the resistance of tissue hydraulics is reduced, and combined with reperfusion, more capillary filtration is carried out, local tissue fluid flows faster, which is more conducive to the recovery of the disease (52). Reperfusion activity refers to the doctor’s use of the other hand or other parts of the body to prompt the patient to rhythmically and significantly or heavily move the muscles related to the needle insertion point within a short period of time, or the patient’s own conscious repeated movement of the muscles related to the disease. The repeated movement of the relevant muscles near the needle insertion point allows the blood to flow back to the ischemic area and also makes the blood flow in a wavy pattern, enhancing the power of the blood flow and expanding its range, thereby improving the ischemic state of the local tissue and relieving spasm and swelling, thereby reducing pain and improving the therapeutic effect (53) (Figure 8). The reperfusion activity can accelerate the blood circulation, so that the blood no longer flows slowly and in small amounts to the ischemic site, but advances strongly, rapidly, and in sufficient quantities to the local area, allowing the ischemic site to receive blood perfusion (54).

In terms of safety, only three studies in this meta-analysis explicitly reported adverse events, while the remaining studies did not mention any safety information. Based on the limited data, the adverse events related to Fu’s subcutaneous needling therapy mainly included subcutaneous bleeding/hematoma (five cases in the Fu’s subcutaneous needling group, four cases in the control group), local pain/discomfort (three cases in the control group), needle body stalling (one case in Fu’s subcutaneous needling group, and one case in the control group), puncture site infection (one case in Fu’s subcutaneous needling group and two cases in the control group), tachycardia (one case in Fu’s subcutaneous needling group and one case in the control group), and skin pruritus (one case in Fu’s subcutaneous needling group). All adverse events were mild and could be relieved spontaneously, and no serious adverse events requiring medical intervention or leading to study withdrawal were observed. The mechanisms of the above adverse reactions are closely related to the operational characteristics of Fu’s subcutaneous needling therapy: ① Subcutaneous bleeding/hematoma is the most common reaction of Fu’s subcutaneous needling therapy. As Fu’s subcutaneous needling performs sweeping operations in the superficial subcutaneous fascia layer, it may stimulate the subcutaneous capillary network, but since Fu’s subcutaneous needling only acts on the subcutaneous layer and avoids deep blood vessels and nerves, the bleeding is usually localized and easy to absorb; ② Needle body stalling may be related to prolonged sweeping time or patient muscle tension, suggesting that the operator should adjust the sweeping intensity according to the patient’s response; ③ The incidence of puncture site infection is extremely low, and there is no significant difference between the two groups, indicating that strict aseptic operation can effectively prevent it; ④ Tachycardia may be related to the patient’s nervousness or acupuncture reaction, and it is a transient reaction occasionally seen in acupuncture treatment. Overall, the safety of Fu’s subcutaneous needling therapy is good, and its superficial operation characteristics avoid the risks of serious complications such as organ damage and pneumothorax that may be caused by traditional acupuncture. However, this study has obvious limitations in the assessment of safety: ① Only three studies reported adverse events, and most studies did not provide safety data. There may be reporting bias - that is, only studies with better safety report adverse events, while studies with adverse events may selectively not report or not publish; ② The collection and definition standards of adverse events in each study were inconsistent, and there was no unified assessment tool and reporting standard, making it impossible to conduct a meta-analysis of data merging; ③ The follow-up period of the included studies was generally short, making it impossible to assess delayed adverse events or the safety of long-term treatment. Future RCTs should routinely record and report all adverse events, regardless of whether they are related to the treatment, and adopt internationally recognized adverse event reporting standards (such as the expanded version of CONSORT harms).

We retrieved the international clinical trial registry^1^ and the Chinese clinical trial registry.^2^ Several clinical trials of floating-needle therapy for musculoskeletal disorders were registered (e.g., NCT03605576, NCT06328153, ChiCTR2300079147, ChiCTR2400079351, ChiCTR2300079324, etc.). This shows that Fu’s subcutaneous needling therapy has great research value and potential.

In addition to statistical significance, clinical significance serves as a crucial indicator for evaluating the value of an intervention. The meta-analysis of this study reveals that the pooled effect sizes of Fu’s subcutaneous needling treatment for lumbar disk herniation are as follows: VAS MD = −1.69 points, JOA MD = 3.97 points, and ODI MD = −7.50 points. To interpret the clinical significance of these values, we compared them with the published minimum clinically important difference (MCID) thresholds. Existing research indicates that there are certain differences in the MCID thresholds among patients with lumbar disk herniation. In terms of pain assessment, the MCID of VAS is typically defined as an absolute improvement of 1.0–2.0 points or a relative improvement of 30–33% (55). The observed 1.69-point improvement in VAS in this study has reached the lower-limit threshold of clinically important improvement. Regarding functional impairment, the MCID of ODI is often defined as an absolute improvement of 12.8–16.5 points or a relative improvement of 30% (56). Although the 7.50-point improvement in ODI in this study is statistically significant, it has not reached the MCID threshold recognized by most studies, suggesting that the clinical benefits of Fu’s subcutaneous needling in improving functional impairment may be limited. There is no unified standard for the MCID of JOA scores in the population with lumbar disk herniation, and the reported range in the literature is approximately 2.5–5.0 points (57). The 3.97- point improvement in this study is at the upper-middle level of this range. The above comparison suggests that Fu’s subcutaneous needling treatment has clear clinical significance in pain relief, while its clinical benefits in improving functional impairment are relatively limited. This finding is consistent with the mechanism of action of Fu’s subcutaneous needling; Fu’s subcutaneous needling can rapidly relieve muscle spasm pain by releasing the affected muscles and improving local blood circulation (52–54), but its effect on improving existing nerve root compression or structural functional impairment may require a longer treatment cycle or combination with other interventions. Future research should focus on the long-term follow-up outcomes of Fu’s subcutaneous needling treatment for intervertebral disk displacement (IDD) to more comprehensively evaluate its clinical value.

Limitations of this study: The methodological quality of the included original studies is relatively low, which directly leads to the downgrading of multiple indicators in the GRADE evidence quality rating. This requires us to be cautious when interpreting the observed efficacy and safety conclusions. Our risk-of-bias assessment shows that there are specific deficiencies in multiple key areas, which may significantly affect the robustness of the conclusions. (1) Although 18 studies claim to have used randomization, only a few studies described the specific sequence generation methods (such as computer-generated random numbers), and only one study clearly mentioned the use of the envelope method for allocation concealment. The remaining studies either used high-risk methods (such as alternating order or date of birth) or had unclear descriptions. Inadequate allocation concealment may lead to selection bias, allowing researchers to predict group assignments, which may exaggerate the treatment effect. (2) Blinding is a major challenge in the included trials. Due to the operational characteristics of Fu’s subcutaneous needling therapy (FSN) (involving visible fanning movements and patients’ active participation in reperfusion activities), only one study implemented single-blinding (blinding only the outcome assessors), and no study achieved double-blinding for both subjects and operators. The lack of subject blinding introduces performance bias, and patients’ expectation effects may affect subjective outcome indicators such as pain scores (VAS) and functional indices (JOA, ODI). Similarly, the lack of observer blinding increases the risk of detection bias, especially for outcome indicators that require subjective judgment. (3) A large number of included studies have small sample sizes (ranging from 30 to 90 cases per group), and none of them reported prior sample size calculations or power analyses. Small samples not only increase the risk of type II errors (failure to detect a true effect) but may also lead to an overestimation of the effect size due to the “small-sample effect.” This imprecision is also reflected in our GRADE assessment, where the evidence quality of VAS and ODI was downgraded due to imprecision and inconsistency. (4) Most included studies mainly focused on short-term outcomes (treatment courses of 7–28 days) and lacked long-term follow-up data. The lack of an extended follow-up period makes it impossible for us to draw definite conclusions about the sustained efficacy of Fu’s subcutaneous needling therapy and potential late-onset adverse events. In addition, if the intention-to-treat (ITT) analysis is not used to handle the high dropout rate in long-term studies, the validity of long - term conclusions will be further compromised. (5) Although the English databases were systematically searched, no English literature meeting the inclusion criteria was found, which reflects the lack of international research on Fu’s subcutaneous needling therapy. The above-mentioned methodological deficiencies indicate that the positive results observed in this study may be overestimated. (6) The included studies showed significant differences in the operational details of floating needle therapy (needle insertion points selection, dispersion time, and re-perfusion activity frequency), treatment duration (ranging from 7 to 60 days), and follow-up time points. This clinical heterogeneity prevented us from determining a unified and reproducible optimal treatment plan. This explains why continuous indicators such as VAS and ODI exhibit high heterogeneity (*I*^2^ > 90%). Specifically: ① Whether the needle insertion points are targeted at the affected muscles and the length of dispersion time directly affect the ‘dose’ of treatment; ② The presence and intensity of re-perfusion activities may alter the treatment effect; ③ The varying treatment duration affects the comparability of efficacy assessment. Although there are differences in operational details, all included studies adopted the core concept of floating needle therapy, and the sensitivity analysis showed a consistent effect direction, which to some extent enhanced the robustness of the conclusion. (7) The meta-analysis of ‘clinical total effective rate’ has certain limitations: Although the efficacy criteria used in each included study were all centered on clinical symptom improvement, the specific criteria for judgment varied (most based on the “Clinical Diagnosis and Therapeutic Effect Standards of Traditional Chinese Medicine”). This difference in judgment criteria may be one of the reasons for the moderate heterogeneity in the effective rate (*I*^2^ = 50%). Nevertheless, all studies’ determination of ‘effectiveness’ pointed to substantive improvement in symptoms, and the sensitivity analysis showed a stable combined effect size, suggesting that this outcome indicator still has clinical reference value. The high heterogeneity observed in the outcomes can be partly attributed to differences in study quality and operation protocols. Therefore, although the existing evidence supports the potential of Fu’s subcutaneous needling therapy, our confidence in these estimates is limited.

To bridge these gaps and guide future research, we propose the following suggestions: (1) future research must strictly adhere to the CONSORT guidelines, adopt robust randomization methods (such as central randomization), and implement strict allocation concealment measures. Although it is difficult to implement double-blinding for operators in acupuncture-related trials, future research should prioritize ensuring the blinding of outcome assessors and statistical analysts. The use of sham acupuncture controls (such as non-penetrating needles or needling at non-trigger points) should be explored to better separate the specific effects of Fu’s subcutaneous needling from placebo effects. (2) Conduct multi-center, large-sample randomized controlled trials with pre-calculated sample sizes to provide sufficient statistical power and external generalizability. (3) Developing a standard operating procedure (SOP) for Fu’s subcutaneous needling therapy (such as needle insertion depth, fanning frequency, and reperfusion activity intensity) is crucial for reducing clinical heterogeneity. (4) Studies should include long-term follow-up periods (such as 3, 6, and 12 months) to evaluate the persistence of treatment effects. Only through such high-quality evidence can the clinical value of Fu’s subcutaneous needling therapy for intervertebral disk displacement be finally established.

Although this meta-analysis concludes that Fu’s subcutaneous needling is superior, it must be interpreted carefully in combination with the significant heterogeneity. First, there is significant clinical heterogeneity: (1) Differences in intervention protocols: Although collectively referred to as “Fu’s subcutaneous needling,” there are differences in the selection of needle insertion points (whether targeting the affected muscles), fanning time, and the standardization of reperfusion activities in each study, which directly affect the “dose” of treatment and may be the core reason for the high heterogeneity of VAS, ODI, etc. (2) Confounding of control measures: The control groups include various methods such as filiform acupuncture, electro-acupuncture, drugs, and rehabilitation. The efficacy of these treatments varies, resulting in different baselines for comparing the “Fu’s subcutaneous needling effect.” Although subgroup analysis was stratified by control measures, there are still differences in acupuncture point selection and techniques within each subgroup (such as the filiform acupuncture group), and the heterogeneity cannot be completely eliminated. (3) Differences in patient populations: The included studies cover IDD patients with different disease durations, herniation types, and severities. Fu’s subcutaneous needling therapy may be more effective for certain subtypes (such as the acute phase or those with mainly muscle spasm), while its effect on patients with severe nerve root compression is limited. Due to the lack of original data in this study, subgroup analysis at the patient level cannot be performed, and this “heterogeneity of individual treatment responses” is submerged in the aggregated data. Future research should focus on reducing heterogeneity: (1) Develop and promote an SOP for Fu’s subcutaneous needling treatment of IDD; (2) Conduct “pragmatic randomized controlled trials” to evaluate the efficacy in the real world under a relatively unified protocol, and the results will be more generalizable; (3) Conduct a meta-analysis of individual patient data to deeply explore the characteristics of the population that benefits most from Fu’s subcutaneous needling treatment.

## 5 Conclusion

This study used meta-analysis to evaluate the clinical Efficacy of Fu’s subcutaneous needling in treating IDD. The results showed that FSN had significant advantages over the control group. Fu’s subcutaneous needling is effective for IDD, and clinicians may consider its use. However, due to the limited number of included studies, more multicenter, large-sample, high-quality RCTs are needed to further validate its clinical efficacy.

## Data Availability

The raw data supporting the conclusions of this article will be made available by the authors, without undue reservation.

## Footnotes

1
https://clinicaltrials.gov/

2
https://www.chictr.org.cn/
