# Supplementary material for: Fu’s subcutaneous needling therapy for intervertebral disk displacement: a systematic review and meta-analysis
Source: Front Med (Lausanne). 2026 Apr 13;13:1781799. doi: 10.3389/fmed.2026.1781799 (PMC13112486; doi:10.3389/fmed.2026.1781799)
Supplement: Supplementary file 1 [file Supplementary_File_1.doc]

**1.Search strategy**

**(1)Embase**

| **Search number** | **Query** |
| --- | --- |
| #1 | Fu’s subcutaneous needling OR Fu’s acupuncture therapy OR float needle[Title/Abstract] |
| #2 | Intervertebral Disc Displacement[exp] |
| #3 | Intervertebral Disc Displacement* OR Protruded Disc* OR Protruded Disk* OR Intervertebral Disk Displacement* OR Herniated Disk* OR  Slipped Disk* OR Disk Prolapse* OR Prolapsed Disk* OR Herniated Disc* OR Slipped Disc* OR Prolapsed Disc* OR Disc Herniation* OR Disk Herniation* OR Disc Protrusion* OR Disk Protrusion*[Title/Abstract] |
| #4 | #2 OR #3 |
| #5 | #1 AND #4 |

**(2)Cochrane library**

| **Search number** | **Query** |
| --- | --- |
| #1 | Fu’s subcutaneous needling OR Fu’s acupuncture therapy OR float needle[Title/Abstract/Keyword] |
| #2 | Intervertebral Disc Displacement[Mesh terms] |
| #3 | Intervertebral Disc Displacement* OR Protruded Disc* OR Protruded Disk* OR Intervertebral Disk Displacement* OR Herniated Disk* OR  Slipped Disk* OR Disk Prolapse* OR Prolapsed Disk* OR Herniated Disc* OR Slipped Disc* OR Prolapsed Disc* OR Disc Herniation* OR Disk Herniation* OR Disc Protrusion* OR Disk Protrusion*[Title/Abstract/Keyword] |
| #4 | #2 OR #3 |
| #5 | #1 AND #4 |

**(3)Web of science**

| **Search number** | **Query** |
| --- | --- |
| #1 | Fu’s subcutaneous needling OR Fu’s acupuncture therapy OR float needle[Topic] |
| #2 | Intervertebral Disc Displacement* OR Protruded Disc* OR Protruded Disk* OR Intervertebral Disk Displacement* OR Herniated Disk* OR  Slipped Disk* OR Disk Prolapse* OR Prolapsed Disk* OR Herniated Disc* OR Slipped Disc* OR Prolapsed Disc* OR Disc Herniation* OR Disk Herniation* OR Disc Protrusion* OR Disk Protrusion*[Topic] |
| #3 | #1 AND #3 |

**(4)CNKI,Wanfang,VIP,SinoMed**

| **Search number** | **Query** |
| --- | --- |
| #1 | Fu’s subcutaneous needling OR Fu’s acupuncture therapy OR float needle[Title/Abstract] |
| #2 | Intervertebral Disc Displacement* OR Protruded Disc* OR Protruded Disk* OR Intervertebral Disk Displacement* OR Herniated Disk* OR  Slipped Disk* OR Disk Prolapse* OR Prolapsed Disk* OR Herniated Disc* OR Slipped Disc* OR Prolapsed Disc* OR Disc Herniation* OR Disk Herniation* OR Disc Protrusion* OR Disk Protrusion*[Title/Abstract] |
| #3 | #1 AND #3 |

**2.Subgroup analysis**

**(1)Efficacy**


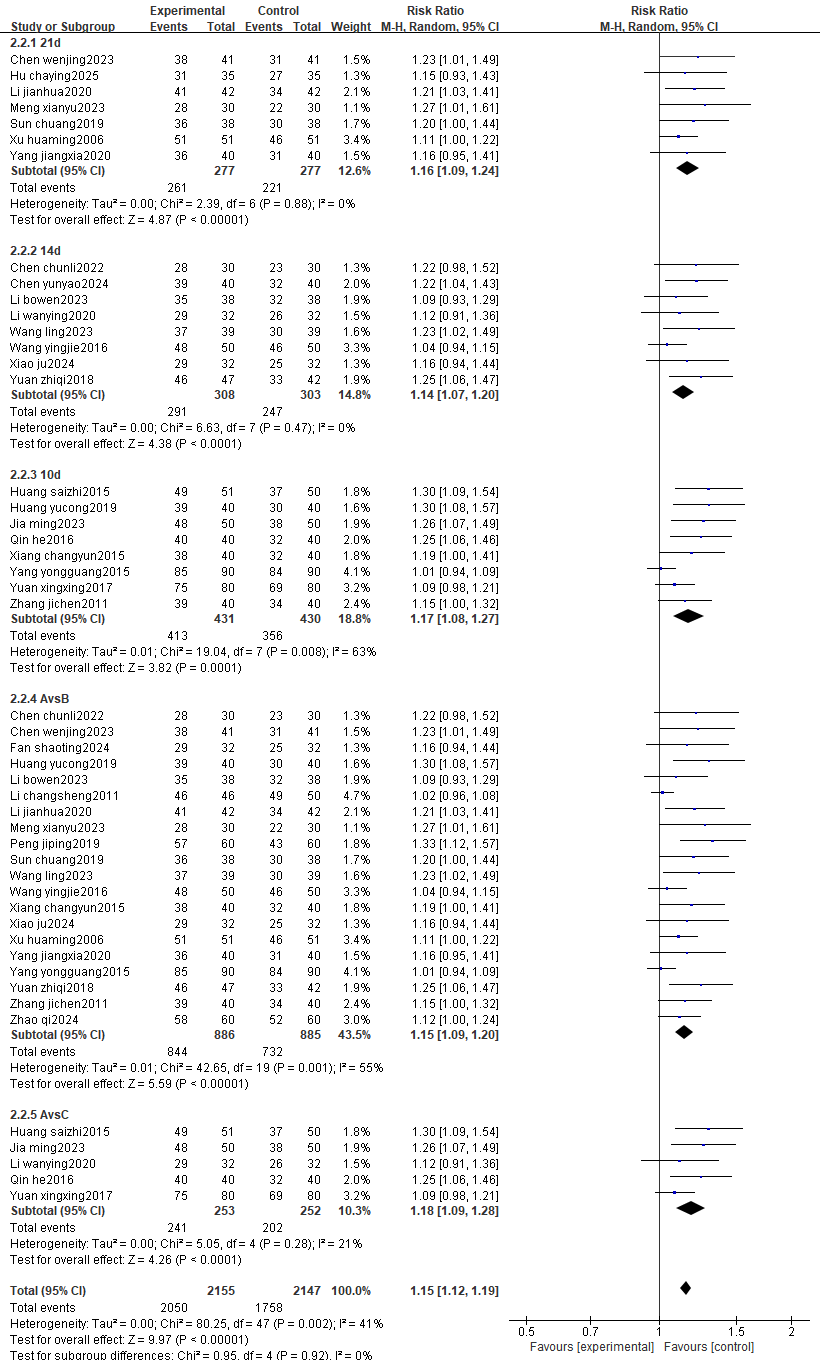


**(2)VAS**


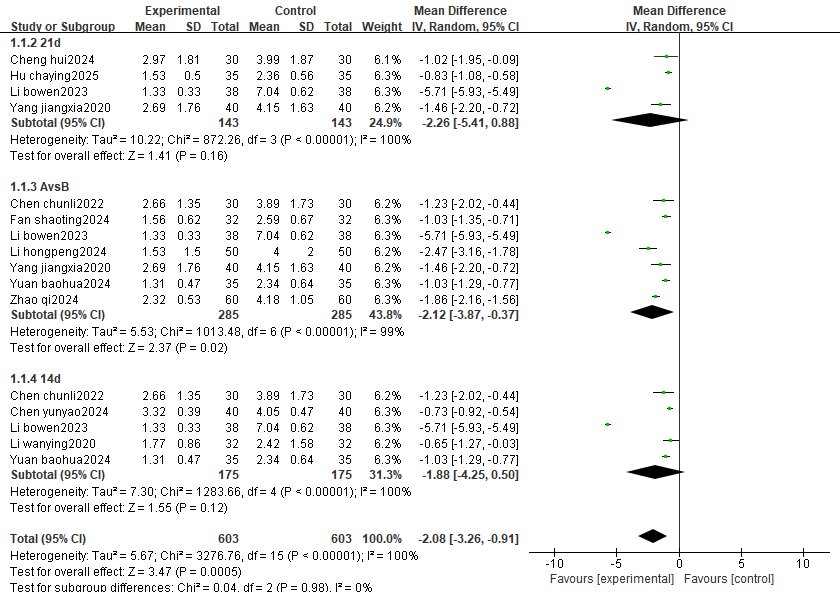


**(3)ODI**


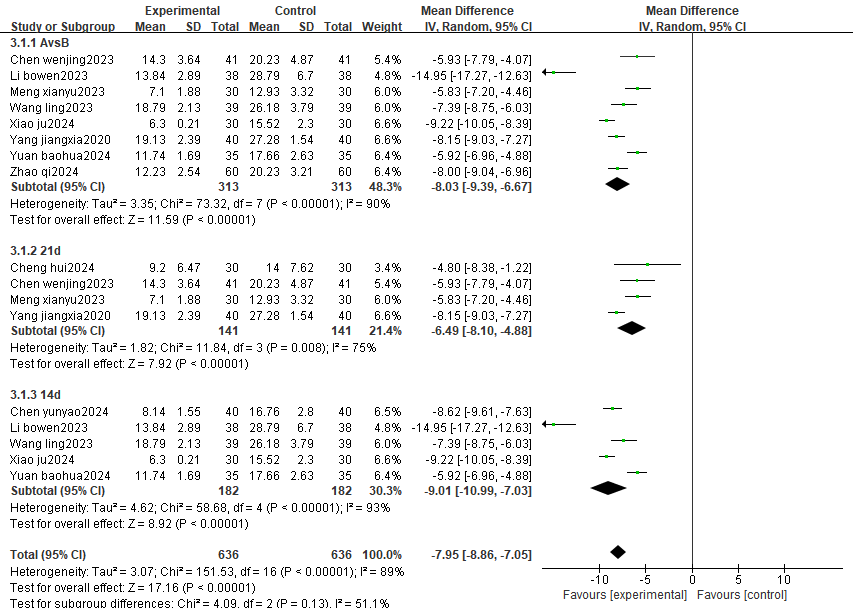


**3.GRADEPro result**


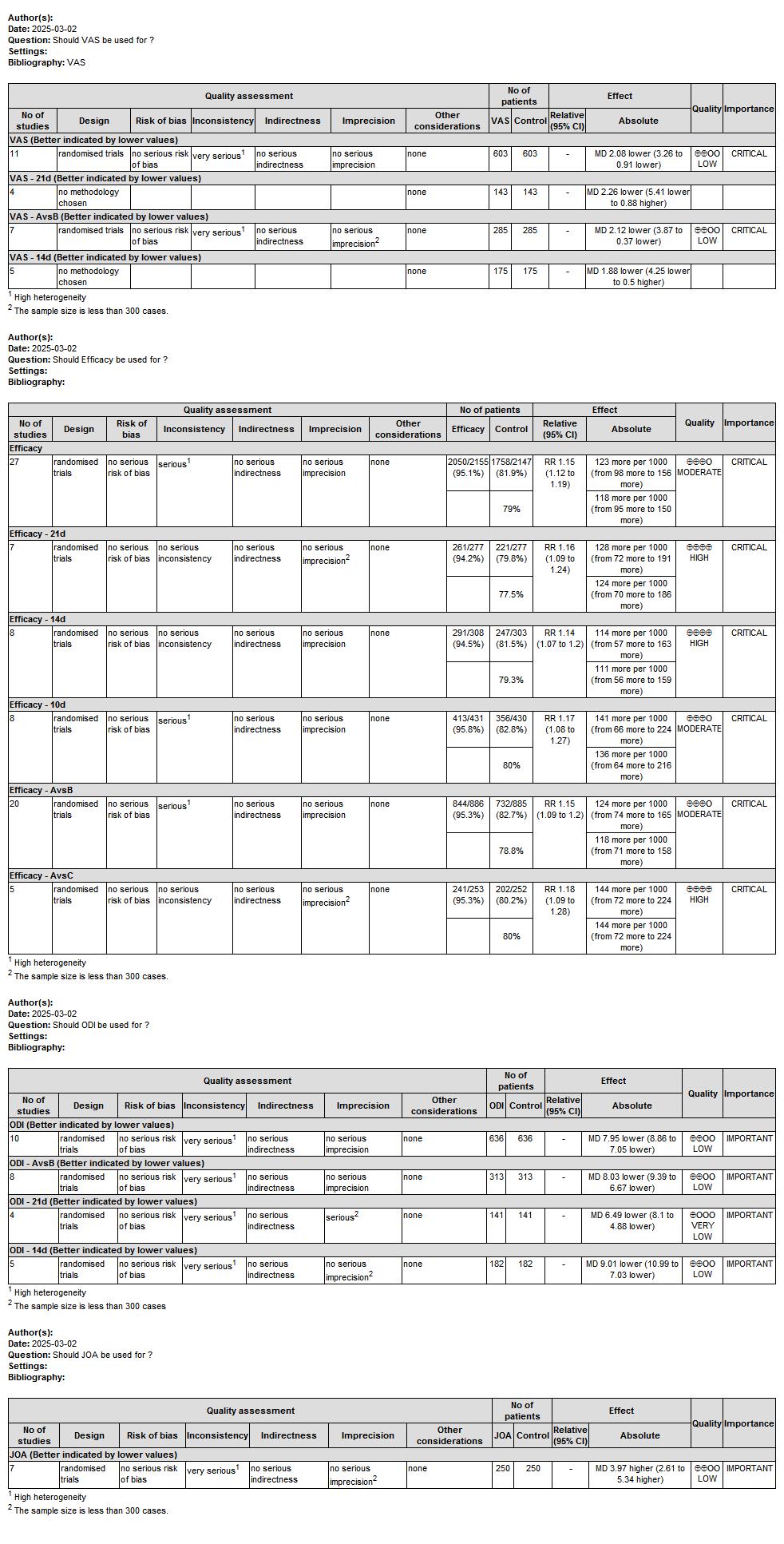


**4.The introduction of Fu's subcutaneous needling(FSN)**

Fu's subcutaneous needling therapy is an invasive non-pharmaceutical treatment method, belonging to a type of needling therapy. It mainly uses disposable FSN devices (referred to as FSNs) to perform sweeping and other needling activities in the loose connective tissue under the skin around localized pain areas.This therapy is based on local symptoms and needles are inserted around the pain (rather than directly at the pain site), with the needle tip aimed at the lesion and the needle body moving along the subcutaneous superficial fascia layer (mainly the loose connective tissue under the skin). Compared with traditional acupuncture methods, it has a longer needle retention time and is mainly used to treat local conditions(The process of needle insertion is shown in the following figure a-b).


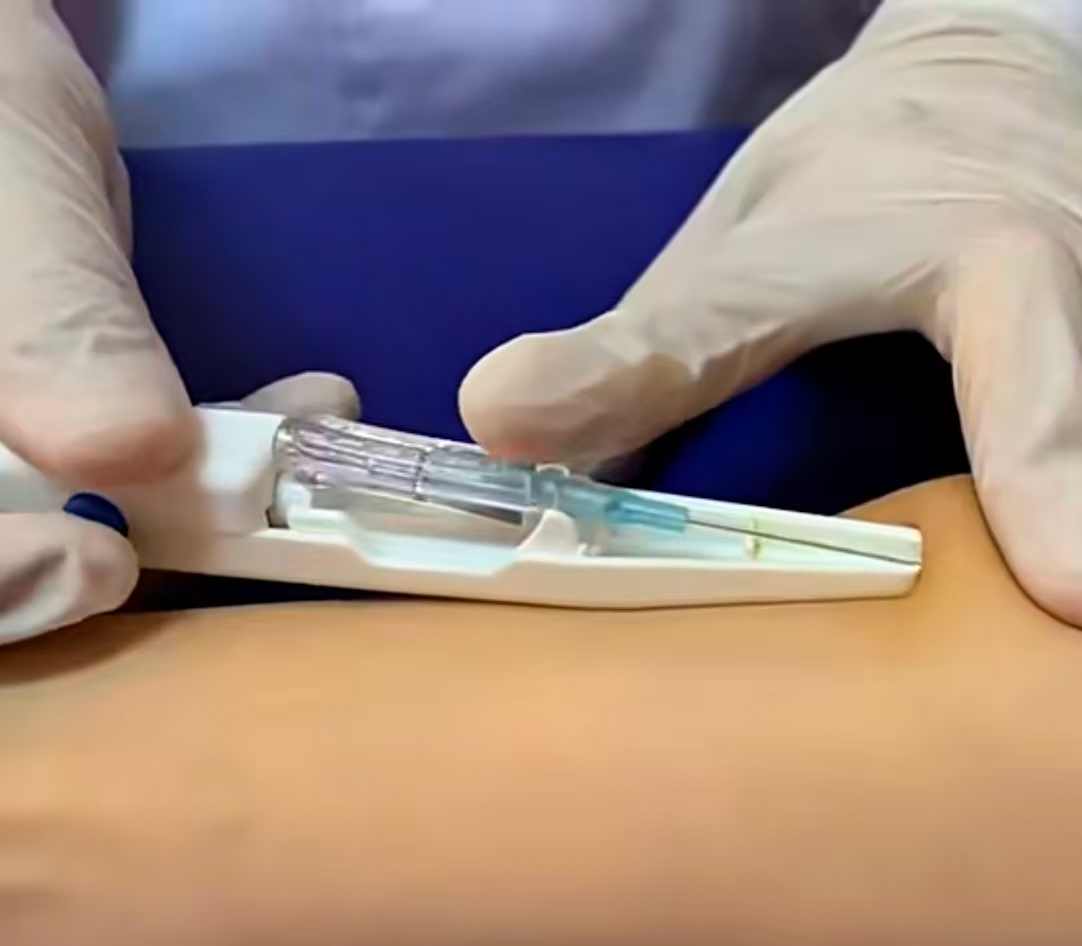


a


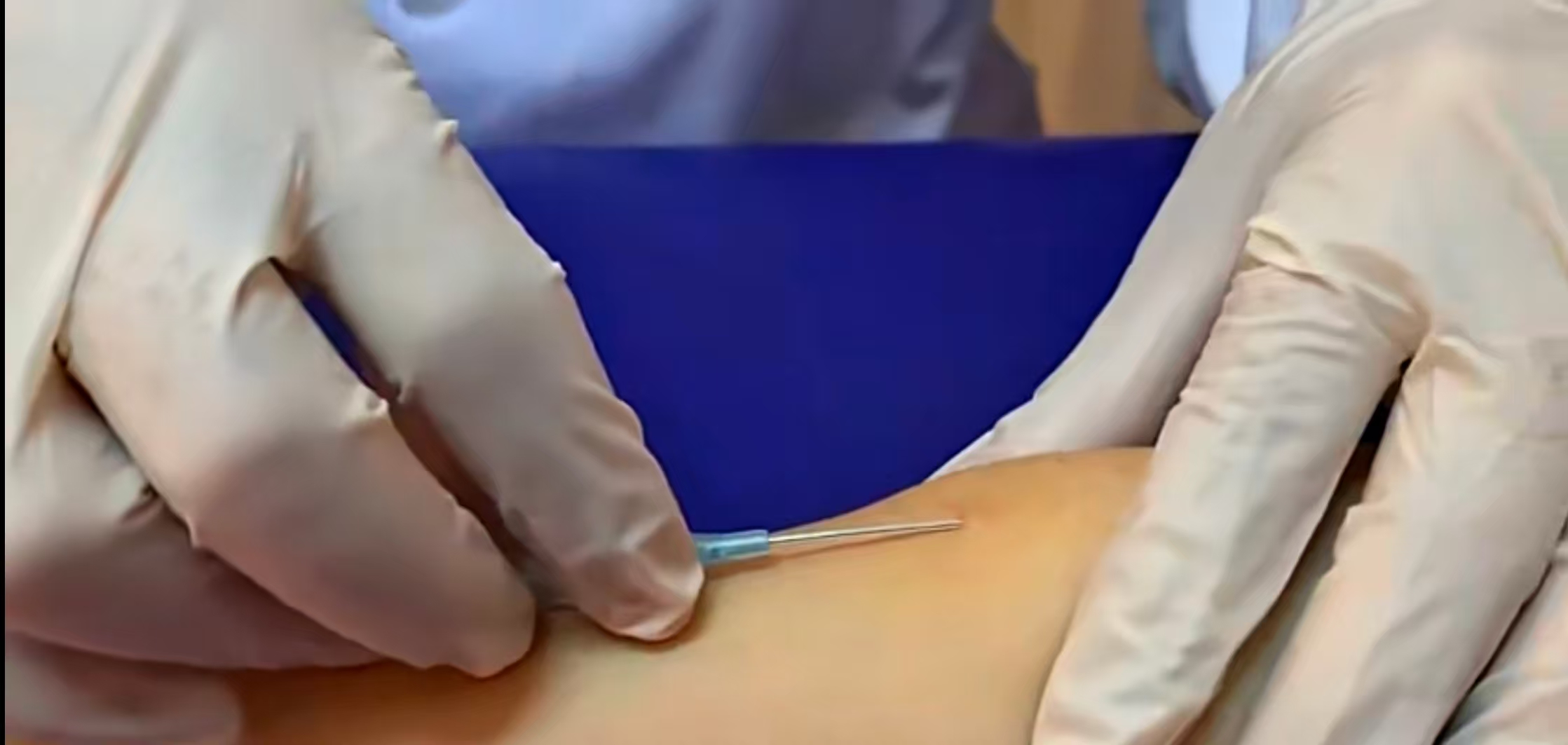


b


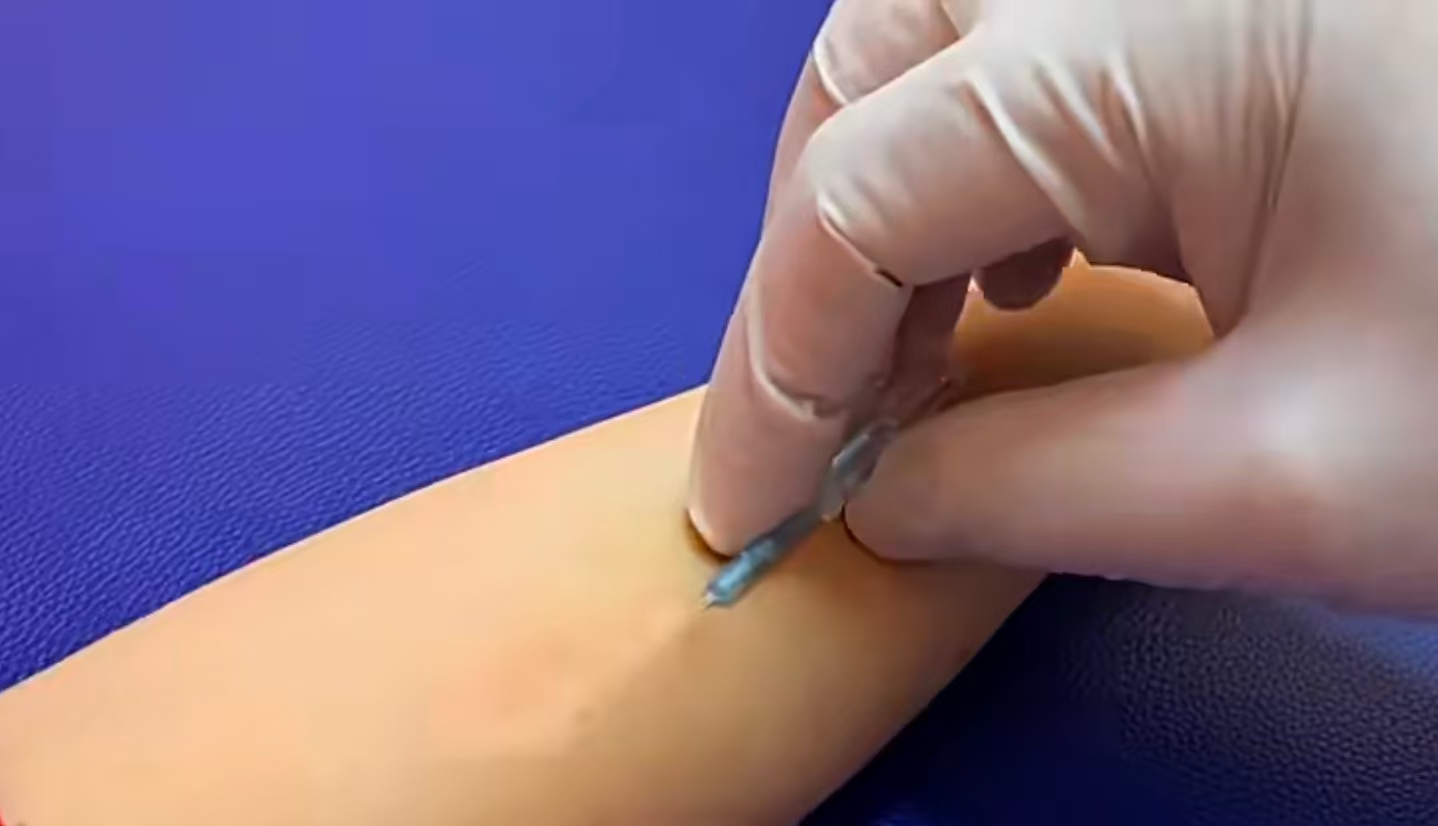


c
